# Extratesticular intrascrotal epidermoid cysts presenting like polyorchidism: a case report

**DOI:** 10.11604/pamj.2024.49.35.44387

**Published:** 2024-10-11

**Authors:** Apidha Kartinasari, Muh Haedar, Yacob Massang, Syarif Syarif, Muhammad Faruk

**Affiliations:** 1 H. Andi Abdurrahman Noor Hospital, Tanah Bumbu, South Kalimantan, Indonesia,; 2 Marina Permata Hospital, Tanah Bumbu, South Kalimantan, Indonesia,; 3 Department of Surgery, Rumah Sakit Umum Dr Daerah H Andi Abdurrahman Noor Hospital, Tanah Bumbu, South Kalimantan, Indonesia,; 4 Department of Urology, Faculty of Medicine, Hasanuddin University, Makassar, Indonesia,; 5 Department of Surgery, Faculty of Medicine, Hasanuddin University, Makassar, Indonesia

**Keywords:** Epidermoid cyst, polyorchidism, testis, ultrasonography, case report

## Abstract

Bilateral scrotal masses may present as polyorchidism or benign neoplasms. Epidermoid cysts (ECs) are common benign cutaneous lesions that are characterized by encapsulated sebaceous cysts containing keratin. These cysts can undergo complications such as ruptures, infections, or daughter cyst formation. A 29-year-old male presented with an asymptomatic scrotal mass. On physical examination, intrascrotal masses were palpated superior to each testis. An ultrasonographic evaluation revealed that the two masses were discrete and located superior to the right and left testis, respectively. The parenchymal echogenicity of these lesions was comparable to that of normal testicular parenchyma. Under the clinical impression of benign scrotal lesions, the provisional diagnosis was of bilateral testicular masses due to polyorchidism. Surgical intervention involved the complete excision of both masses. The subsequent histopathological examination revealed the definitive diagnosis of ECs. This case illustrates that despite the advantages of Doppler imaging, ultrasonography may yield less accurate results than histopathological findings.

## Introduction

Epidermoid cysts (ECs) are the most prevalent type of benign epithelial cyst and generally have minimal malignant potential [1]. While ECs are predominantly intratesticular, they can present in extratesticular locations. Genital extratesticular cysts are exceedingly rare and typically manifest as discrete, painless masses occurring anywhere along the genitourinary tract from the distal penile frenulum to the anal canal [2]. Although rare, ECs constitute the second most common benign testicular neoplasms, with peak incidence occurring between the ages of 20 and 40. The most frequent clinical presentation is a palpable, painless testicular mass, which may raise suspicion of malignancy [1]. Testicular masses occasionally indicate numerical abnormalities of the testes, such as polyorchidism, which is an exceedingly rare condition with fewer than 250 reported cases in the literature. Polyorchidism, or supernumerary testis, is a congenital anomaly of the genitourinary system characterized by the presence of more than two testes [3]. In this study, we report an unusual case of bilateral scrotal ECs mimicking polyorchidism.

## Patient and observation

**Patient information:** a 29-year-old Asian man complained of painless lumps in both testicles over a period of two years. Both lumps had increased in size and their consistency became firm. Two years ago the masses were the size of a marble; however, they increased and the patient now felt that both were the size of bottle caps. The lumps were not painful when held or pressed. The complaint was not accompanied by significant recent weight loss or problems with urination or intercourse. There was no history of fever, lymphangitis, lymphadenitis, infertility, fatigue, dyspnea, trauma, malaise, loss of consciousness, or previous testicular tumors. In addition, the patient had no history of stones in the urine or hematuria. No previous accounts of allergies to food, medicines, or dust were noted. Although the patient had hypertension, no regular treatment had been administered. The patient had no history of diabetes mellitus and no similar diseases were observed within his family.

**Figure 1:**
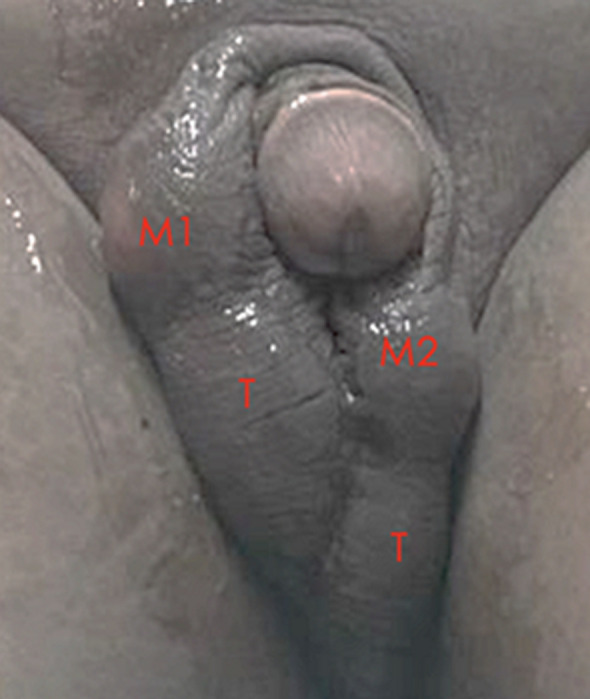
macroscopic appearance of intrascrotal masses

**Clinical findings:** on physical examination, the penis structure was normal with two lumps above the scrotum. The palpable right intrascrotal mass (M1) measured 4x3x2.5 cm and was identified as a skin-colored lump above the right testicle with a flat surface and soft consistency that was mobile and without tenderness. The left intrascrotal mass (M2) measured 3x3x1.5 cm and was characterized as a skin-colored lump above the left testicle with a flat surface and soft consistency that was mobile and without tenderness (Figure 1). Palpation of the bilateral testes and spermatic cord revealed no extraordinary abnormalities. The transillumination tests for both masses were negative. There was no inguinal lymphadenopathy.

**Figure 2:**
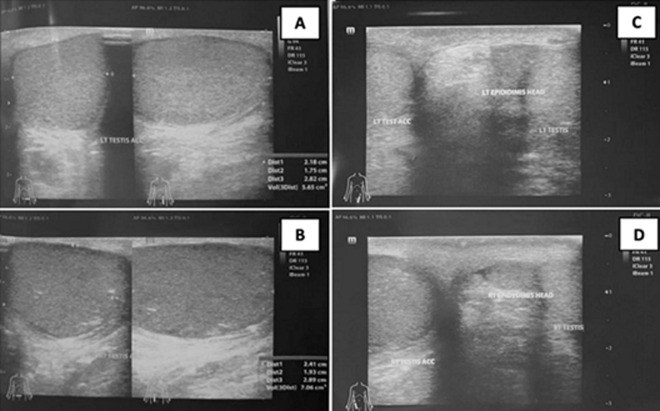
ultrasonography of scrotum showing: A) left and right; B) intrascrotal masses; C) the left and right; D) epididymides were within normal limits

**Diagnostic assessment and therapeutic intervention:** the laboratory examination showed that the complete blood count and the liver and kidney function tests were within normal limits. The scrotal ultrasound showed supernumerary testicles with accessory testicles on the left and right superior testicles. The ultrasound revealed two masses with the same parenchymal structure as the right (volume ± 7.1 cm^3^) and left (volume ± 5.6 cm^3^) superior testis. The parenchymal structure was homogeneous, showing no intralesional vascularization with color Doppler. The left and right epididymis were within normal limits. No fluid collection was seen in the left or right peritesticular region (Figure 2). The diagnosis of bilateral testicular masses due to polyorchidism was established. Subsequently, a bilateral testicular tumor orchiectomy was performed by a general surgeon in the operating room and the specimen was sent to the pathology anatomy department.

**Figure 3:**
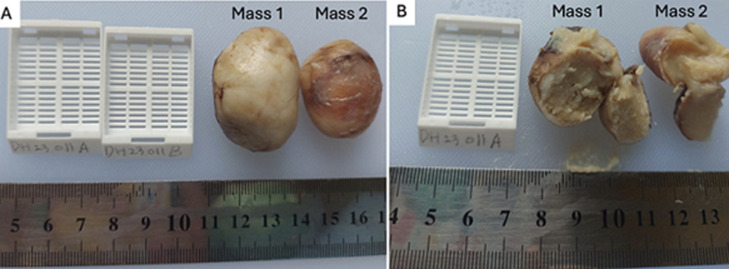
macroscopic histopathological examination

**Figure 4:**
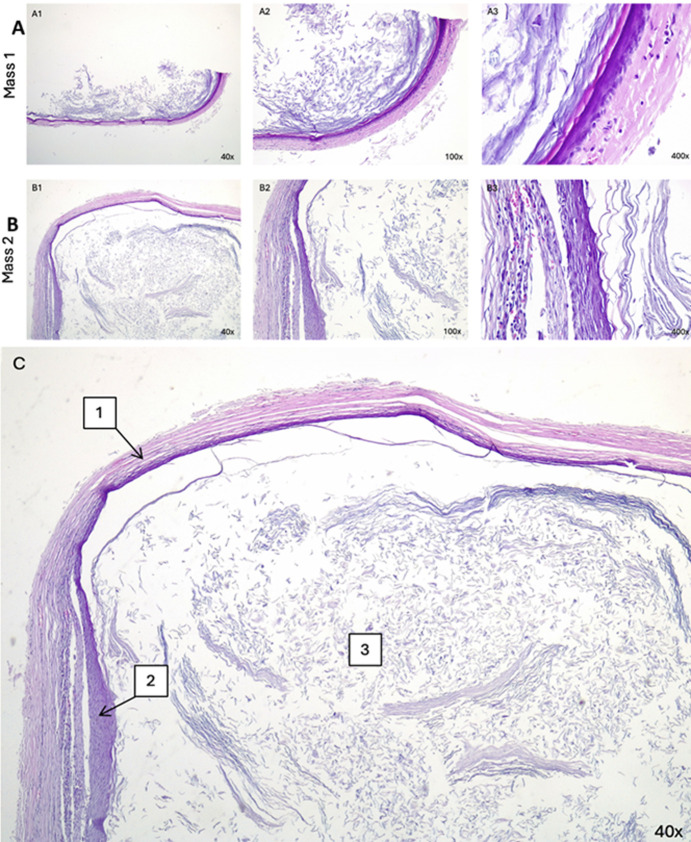
A, B) microscopic histopathological examinations of mass 1 and mass 2 at magnifications of 40x, 100x, and 400x (A1-3 and B1-3, respectively); C) showing the histopathology by layer; 1) the cyst wall containing connective tissue, blood vessels, and lymphocyte inflammatory cells; 2) the lining of the cyst wall composed of stratified squamous epithelium; 3) and the lumen of the cyst with a mass of lamellar keratin

**Follow-up and outcome of interventions:** the macroscopic histopathological examination identified a right scrotal mass (M1) measuring 3.8x3x2.8 cm and a left scrotal mass (M2) measuring 3.2x2.5x1.7 cm with smooth outer surfaces, brownish cream colors, and palpable cystic consistencies. Cross-sections of the slices revealed a yellowish-white mass (Figure 3). Microscopically, both tissue preparations had a cyst wall lined with a stratified squamous epithelium with a non-atypical nucleus and a mass of lamellar keratin inside the lumen. There were no seminiferous tubules in the preparation (Figure 4). The definitive diagnosis of bilateral scrotal masses due to ECs was established. The patient returned to an outpatient department 10 days after discharge and no complications were found.

**Patient perspective:** the patient felt better in conducting daily activities after discharge from the hospital.

**Informed consent:** the patient provided informed consent regarding the publication of this case report.

## Discussion

Epidermoid cysts (ECs) are benign neoplasms that comprise 1-2% of all testicular neoplasms and are characterized by keratin-filled cavities [4]. Epidermoid cysts can present as unilateral, bilateral, or multifocal lesions and may occur anywhere along the midline from the cranium to the anal midline [5]. The median age of presentation and mean lesion diameter were recently reported as 26 years (range 11-34 years) and 1.74 cm, respectively [4]. The etiology may involve the aberrant embryological closure of the neural groove or epithelial fusion planes [5], in which case, bilateral ECs manifest as soft, mobile, and non-tender masses. Most cases present as asymptomatic masses that may suggest malignancy, particularly in young adults and patients with a predominance of right-sided involvement [4]. In the evaluation of testicular masses, differentiation between benign and malignant pathology is crucial to avoid an unnecessary orchiectomy. This poses a clinical dilemma for both the patient and urologist in terms of testicular preservation when the lesion is intratesticular. In the current case, a physical examination revealed two asymptomatic scrotal masses that mimicked testicular tissue. Scrotal ultrasonography is typically the initial and most critical modality for evaluating scrotal contents as this technique enables the differentiation of intratesticular and extratesticular lesions and the characterization of the lesions as cystic or solid [1]. The sonographic appearance of intratesticular lesions may present an “onion skin” pattern, which is formed by the cyst enlarging within the testis and outwardly displacing the surrounding parenchyma. In contrast, extratesticular lesions often appear as well-circumscribed masses, with variable echotexture ranging from hypoechoic to hyperechoic depending on their composition [6].

In our patient, the scrotal ultrasonography revealed a homogeneous, hypoechoic, discrete, extratesticular mass without intralesional vascularity on color Doppler imaging. The absence of vascularization or identifiable epididymal tissue within the mass suggested supernumerary testis or polyorchidism type B2 according to the Bergholz classification, which is based on the presence or absence of drainage to the vas deferens (VD). Type A exhibits VD drainage and is further subdivided as follows: A1 has a separate epididymis and VD, A2 has an epididymis but shares the VD with another testicle, and A3 shares both the epididymis and VD with another testicle. Type B lacks VD drainage and is divided into B1 (separate epididymis) and B2 (no epididymis) [3,7]. Polyorchidism is associated with various urogenital anomalies including inguinal hernia, cryptorchidism, hydrocele, testicular torsion, and testicular neoplasms [8]. Further diagnostic evaluation may include the assessment of testicular malignancies using serum tumor markers such as lactate dehydrogenase (LDH), human chorionic gonadotropin (hCG), and alpha-fetoprotein (AFP) [8]. Magnetic resonance imaging (MRI) can provide complementary information when the ultrasonographic findings are indeterminate [9]. However, due to the risk of malignancy, a definitive diagnosis of scrotal masses often requires histopathological examination [8]. The Doppler ultrasound found no vascularization of the cyst core. In ECs case, an MRI that confirmed no signal enhancement when contrast was given to the cyst core could indicate that the cyst was avascular [3].

Our facility lacked MRI capabilities and we opted for a post-operative biopsy. The sonographic features characteristic of polyorchidism did not exclude other testicular pathologies such as ECs or malignant neoplasms such as liposarcoma, fibrosarcoma, or metastatic disease; therefore, complete surgical excision of the extratesticular cyst was performed and the specimen was sent for a pathological examination to confirm the diagnosis. Histopathological examination of ECs typically reveals a well-defined cyst lined by a fibrous membrane and filled with laminated keratin and cellular debris [4]. In our case, the cyst walls of both masses were lined with stratified squamous epithelium with non-atypical nuclei and lumina containing lamellated keratin. The avascular nature of the cysts was confirmed by the absence of a Doppler signal on ultrasonography, consistent with the lack of contrast enhancement on MRI, which is characteristic of ECs. Six months after the tumors were completely removed, the patient was examined and no recurrences were detected in his scrotum. Another study had similar results, in which no recurrent cases were detected during follow-up periods ranging from 12 to 216 months [10].

## Conclusion

In this study, we reported a case of a testicular mass that was diagnosed as polyorchidism based on ultrasonography, successfully resected while preserving the testis, and rediagnosed as EC after histopathological analysis. This case revealed that supporting an examination with Doppler ultrasound may be less accurate than histopathological testing.
